# Supplementary material for: MetaRibo-Seq measures translation in microbiomes
Source: Nat Commun. 2020 Jun 29;11:3268. doi: 10.1038/s41467-020-17081-z (PMC7324362; doi:10.1038/s41467-020-17081-z)
Supplement: Supplementary file 10 — Supplementary Data 7 [file 41467_2020_17081_MOESM10_ESM.zip › File2/Confidence_High_Taxonomy/13737_out.krona.html]

Javascript must be enabled to view this page.

members
magnitude
magnitudeUnassigned
count
unassigned
taxon
rank

13737\_out

2

superkingdom
2
2

2
1239
phylum

1
526524
class

526525
1
order

family
1
128827

genus
1
61170


SRS015264\_contig\_number\_contig-100\_11279.11279
1
1852366
species

species

SRS047433\_contig\_number\_contig-100\_2764.40114
1
1263003
